# Affinity maturation pathway of an anti-MPER neutralizing mAb, CAP206-CH12

**DOI:** 10.1186/1742-4690-9-S2-P65

**Published:** 2012-09-13

**Authors:** NL Tumba, ES Gray, BE Lambson, SS Abdool Karim, H Liao, BF Haynes, M Alam, L Morris

**Affiliations:** 1 NICD- HIV/STI Center, Johannesburg, South Africa; 2 Centre for the AIDS Programme of Research in South Africa (CAPRISA), Durban, South Africa; 3 Duke Human Vaccine Institute, Durham, NC, USA

## Background

The membrane proximal external region (MPER) of HIV-1 is an important target of broadly cross-reactive mAbs. CAPRISA participant, CAP206, developed anti-MPER antibodies early that became cross-neutralizing at 18 months post-infection. This coincided with neutralization of the C4 HIV-2/HIV-1 chimera containing the W670 residue, suggesting changes in an antibody paratope may have resulted in the acquisition of breadth. A neutralizing mAb (CAP206-CH12) was isolated, providing an opportunity to determine which somatic hypermutations contribute to breadth.

## Methods

The putative CAP206-CH12 reverted unmutated ancestor (RUA) was inferred using SoDA (http://www.dulci.org). Batch transient transfections were used to generate recombinant antibodies. Neutralization breadth and potency was tested against autologous, Tier 2, and HIV-2/HIV-1 MPER chimeric viruses using the TZM-bl assay. Binding to MPER peptides was assessed by ELISA and surface plasmon resonance (SPR).

## Results

CAP206-CH12_RUA bound the MPER.03 peptide with a Kd of 120nM, 15-fold weaker than CAP206-CH12 binding, but had no neutralizing activity. Since CAP206-CH12 and its RUA differed by 19 residues in the heavy chain and 9 in the light chain, we designed an intermediate precursor (IP) where changes near the CDRs in CAP206-CH12 were reverted back to the germline sequence (11 in the heavy and 5 in the light chain). This CAP206-CH12_IP did not neutralize the C4 chimera suggesting that changes responsible for the affinity-matured CAP206-CH12 neutralizing capacity were among these 16 residues. Chimeric pairs of the light chain IP with the heavy chain of CAP206-CH12 or CAP206-CH12_RUA showed binding to MPER with differences in binding kinetics.

## Conclusion

The reduced binding and neutralizing activity of CAP206-CH12_RUA and CAP206-CH12_IP compared to CAP206-CH12 suggests a correlation between affinity maturation, neutralization breadth and potency. Ongoing work will assess the affinity maturation of CAP206-CH12 by determining moieties in the antibody paratope associated with effective epitope recognition and the effects of somatic mutations on the evolution of neutralization.

